# Four Remarkable Additions to the Biodiversity of Chinese Mosses

**DOI:** 10.3390/plants11192590

**Published:** 2022-09-30

**Authors:** Mamtimin Sulayman, Vladimir Fedosov, Vítězslav Plášek

**Affiliations:** 1 Xinjiang Key Laboratory of Biological Resources and Genetic Engineering, College of Life Science and Technology, Xinjiang University, Urumqi 830046, China; 2 Department of Biology, Lomonosov Moscow State University, 119234 Moscow, Russia; 3 Botanical Garden-Institute, FEB RAS, Makovskogo Street 142, 690024 Vladivostok, Russia; 4 Department of Botany, University of Ostrava, Chittussiho 10, 710 00 Ostrava, Czech Republic; 5 Institute of Biology, University of Opole, Oleska 48, 45-052 Opole, Poland

**Keywords:** Asia, bryophytes, China, distribution maps, Musci, phytogeography, *Schistidium*, Xinjiang Uyghur Autonomous Region, checklist, identifying key

## Abstract

Four species of moss genus *Schistidium* are reported for the first time from China. All of them have been found in the Xinjiang Uyghur Autonomous Region. Ecological and distributional details of the newly recorded species are provided and their local distribution is mapped. Photographs of the species are attached. Checklist of *Schistidium* species and identifying key are added. Considering the present records, *Schistidium* consists of 15 species in China.

## 1. Introduction

The genus *Schistidium* (Grimmiaceae, Bryophyta) represents an example of outstanding diversity, which was neglected until thorough morphological revision of *S. apocarpum* group by Blom [1]. This revision resurrected from oblivion a number of already existing names and 13 species were described as new for science. Moreover, this revision created a frame, which allowed further increase in the formally recognized biodiversity within the genus by means of regional alpha-taxonomic studies. In the course of such studies, a number of *Schistidium* species were described from Europe [2,3], North America [4,5,6] Russia [7,8] and China [9,10,11], as well as from North Africa [12] and South America [13]. Molecular data [7,14] supported the narrow species concepts of suggested by Blom [1].

Cao and Vitt [15] revised genera *Schistidium* and *Grimmia* and recognized six species of *Schistidium* there (including *Schistidium apocarpum* (Hedw.) Bruch & Schimp., *S. liliputanum* (Müll. Hal.) Deguchi, *S. rivulare* (Brid.) Podp., *S. strictum* (Turner) Loeske ex Mårtensson, *S. subconfertum* (Broth.) Deguchi, and *S. trichodon* (Brid.) Poelt), as well as supported *Schistidium* as an independent genus in Grimmiaceae. Cao et al. [16] transferred *Grimmia chenii* S.H. Lin, known from Xizang and Xinjiang Provinces, into the genus *Schistidium* as *S. chenii* (S.H. Lin) T. Cao, C. Gao & J.C. Zhao. One next species *S. sinensiapocarpum* (Müll. Hal.) Ochyra, described from China (Shaanxi Province) already in 1898 by Carl Müller [17], was considered as a synonym of *S. strictum* by Cao et al. [18] and was not listed by them as separate species in the Moss flora of China. However, these two species are again distinguished now [19]. On the other hand, both Blom [1] and Ignatov [19] agree that *S. strictum*, formerly considered widespread throughout the Northern Hemisphere, occurs only in territory of Great Britain, Iceland, western Scandinavia, on the west coast of North America and very rarely also in the Pyrenees and Madeira. The occurrence of the species in China is therefore very unlikely and it is necessary to remove it from the Chinese checklist.

Recently, Blom et al. [9] reported two *Schistidium* species from China: *S. mucronatum* H.H. Blom, Shevock, D.G. Long & Ochyra and *S. riparium* H.H. Blom, Shevock, D.G. Long & Ochyra. Feng et al. [11] added one more, *S. ignatovae* C. Feng, X.L. Bai, J. Kou & W. Li. Finally, *S. lancifolium* (Kindb.) H.H. Blom was found during field studies in the Inner Mongolia province [20] that increased the number of Chinese *Schistidium* species to eleven.

The data presented above together with four newly reported taxa confirm the presence of fifteen *Schistidium* species in China.

## 2. Materials and Methods

### 2.1. Ecological and Phytogeographic Characteristics

Xinjiang Uyghur Autonomous Region occupying about 1/6 of the national territory, is the largest province of China [21]. It is located in the northwestern boundary of China with a total land area of 1,657,549 km^2^. From north to south there are three mountain ranges across Xinjiang, namely, Altai, Tianshan and Kunlun, with the geomorphologic features being characterized by two basins called the Junggar Basin and the Tarim Basin among three mountains. Therefore, the geographical and geomorphologic features are very complex and diverse in Xinjiang Province [21].

Xinjiang Province is situated in the hinterland of the Asia-Europe continents and is far away from the oceans. In addition, the Himalaya Range blocks the warm moist air currents from the Indian Ocean to arrive in Xinjiang, which is called the “blocking effect” [22]. Just as the causes above, the precipitation is very low and the plant distribution is also uneven (mostly concentrated in the mountains), resulting in the formation of a typical arid and semi-arid climate. Hydrologically, in general, there is a progressive increase in precipitation from south to north, in detail, the average annual precipitation of the basin is about 10–100 mm, that of the mountain is 250–530 mm, while the annual evaporation of the southern border is 1000–1550 mm, that of the north for 1000–1200 mm.

Bryophytes are mainly distributed in the three mountain ranges, so it is necessary to describe the three mountains’ geography, hydrology and vegetation [21,22]. Because of the special environment and topography, Xinjiang has a unique flora representing a mixture of circum-boreal, Irano-Turanian and Eastern Asiatic taxa [21,22]. The vegetation of Altai and Tianshan has apparent vertical distribution belts caused by climate and topography, including desert, steppe zone, mixed conifer, coniferous forest, sub-alpine meadow belt, alpine meadow and alpine vegetation zones. Compared with those of Altai and Tianshan, the Kunlun Mountains lack forest zones, and the distribution belts of the desert and steppe of mountains are rather broad [23,24].

### 2.2. Materials

Totally, 651 specimens of *Schistidium* species were collected by Mamtimin Sulayman and his research team in Xinjiang province during the last 30 years. They mainly explored mountain ranges of the western part of China, particularly Altai Mts, Tianshan Mts, Kunlun Mts and Pamir plateau.

All materials are located in the herbarium of Xinjiang University, China (XJU) and partially also in the herbarium of the University of Ostrava, Czech Republic (OSTR).

The revision of all these collected materials made by authors during 2020–2022 showed that four of the *Schistidium* species were found for the first time in China.

For the identification of the collected material, mainly Moss flora of China, Volume 3 [18] and Moss flora of Russia, Volume 2 [19] were used.

Detailed photo documentation was taken for each of the presented species. The pictures show only material from the herbarium specimens listed below—see captions near each image for details. The extent of quality of the photographs reflects sometimes the condition of the collected material, which was sometimes slightly damaged—but not so much that it could not be reliably identified.

## 3. Results

### 3.1. New Species for China

During bryological expeditions carried out in Xinjiang province during the last 30 years, an extensive collection of *Schistidium* specimens was collected by M. Sulayman and his research team. Four of the species, *Schistidium flaccidum*, *S. marginale*, *S. pulchrum*, and *S. sibiricum* have not hitherto been recorded in China.

**Figure 1:**
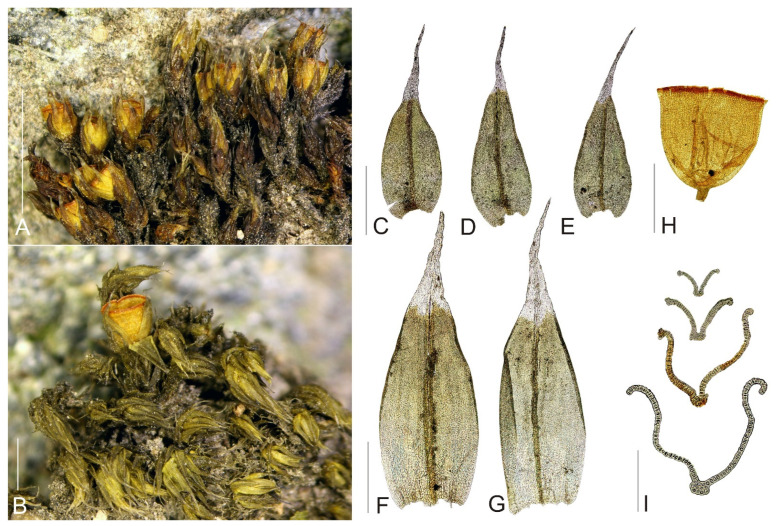
Macro and micro photographs of *Schistidium flaccidum*: (**A**,**B**) view on fertile plants; (**C**–**E**) leaves; (**F**,**G**) perichaetial leaves; (**H**) capsule; (**I**) leaf cross-sections (from the base of the leaf to the tip). Scale bars: (**A**,**B**)—1 mm, (**C**–**H**)—0.5 mm, (**I**)—0.1 mm. All photos from specimen OSTR #7250, made by V. Plášek.

***Schistidium*** ***flaccidum*** (De Not.) Ochyra—Figure 1, and map: Figure 5 (spot 1).

*Schistidium flaccidum* is characterized by long and broad-based awns, light-colored capsules with red rims, a rudimentary peristome, and a short mamillate rostrum.

Description: plants olivaceous or light brown, from 0.5 cm till 2 cm high; leaves erect or weakly curved when dry, ovate-lanceolate, strongly keeled; capsule orange-brown, cupulate, from 0.6 mm till 1 mm long. Capsules are produced from late spring to early summer. This species is characterized by small plant size, perichaetial leaves remarkably larger than lower leaves, plicate, rather weak, not decurrent hyaline hair point, short, cupulate capsule with strongly reduced peristome and flat operculum with only short rostrum.

Ecology: mainly on rocks in open to shaded habitats; occasionally also on an anthropogenic substrate [19].

Specimen examined. Xinjiang Uyghur Autonomous Region. Mulei Co.: Tianshan mountains, 150 km E of Urumqi, ca. 1471 m a.s.l., 44.156389° N, 91.068333° E, leg. M. Sulayman s.n., 26 June 2018 (herb. XJU, OSTR #7250).

*Schistidium flaccidum* is a generitype of the genus *Schistidium*. It has disjunctive distribution, largely associated with Europe, where it reaches Caucasus Eastwards and Western North America with few scattered localities in North Africa and GBIF database [19,25]. Until the present, this species has been known in Asia from a single locality in Transcaucasia two collections in Russian Altai and one recent collection from Tyva Republic, Russia [19,26]. In Siberian localities, it grows on dry rocks in the subalpine to lower alpine belts at elevations 2200–2450 m.

**Figure 2:**
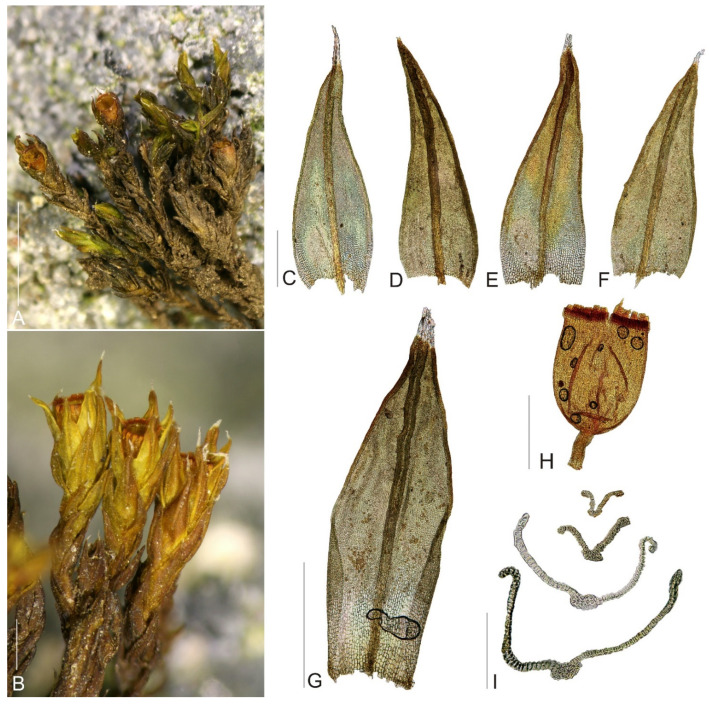
Macro and micro photographs of *Schistidium marginale*: (**A**,**B**) view on fertile plants; (**C**–**F**) leaves; (**G**) perichaetial leaf; (**H**) capsule; (**I**) leaf cross-sections (from the base of the leaf to the tip). Scale bars: (**A**)—1 cm, (**B**)—1 mm, (**C**–**F**)—0.5 mm, (**G**)—1 mm, (**H**)—0.5 µm, (**I**)—0.1 mm. All photos from specimen OSTR #7251, made by V. Plášek.

***Schistidium*** ***marginale*** H.H. Blom, Bednarek-Ochyra & Ochyra—Figure 2, and map: Figure 5 (spot 2).

*Schistidium marginale* has Eurasian distribution from central Europe, where it was described in Russian Far East. In Asia, it is known to occur in Georgia, Turkey and throughout the Asian part of Russia. Within the latter, the species is rather frequent in Transbaikalia, scattered localities with few scattered localities in Putorana Plateau, Eastern part of Yakutia, Altai, Kuznetsky Alatau and Tukuringra mountains [19,26]. In the south Siberian mountains it typically occurs in the forest belt, occupying an altitudinal range of 600–1700 m, on shaded siliceous rocks [19,26].

Description: plants small, dull or brownish above, from 1 to 2.5 cm high, leaves densely set, shiny, erect, narrowly ovate-triangular, keeled; Sporophytes greyish-brown, shiny, obloid-cylindrical, becoming finely striate with age, from 0.6 mm to 1 mm long.

This species is characterized by small plant size, keeled distal portions of leaves with short and strongly denticulate hyaline own, costae irregularly angulate on the cross-section and strongly incrassate 2–4 layered distal leaf margins [19].

Ecology: grows mainly on inclined rock ledges [19].

Specimen examined. Xinjiang Uyghur Autonomous Region. Mulei Co.: Tianshan mountains, 200 km E of Urumqi, ca. 1567 m a.s.l., 44.093889° N, 90.089722° E, leg. M. Sulayman s.n., 27 June 2018 (herb. XJU, OSTR #7251).

**Figure 3:**
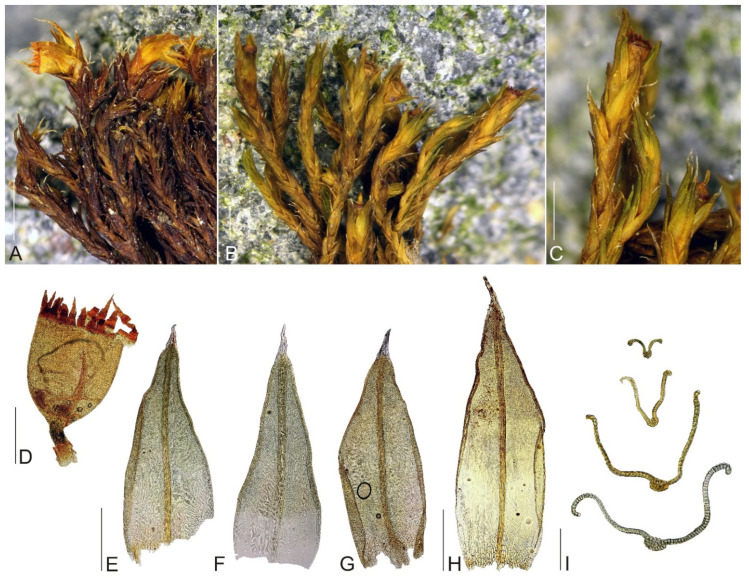
Macro and micro photographs of *Schistidium pulchrum*: (**A**–**C**) view on fertile plants; (**D**)—capsule; (**E**–**G**) leaves; (**H**) perichaetial leaf; (**I**) leaf cross-sections (from the base of the leaf to the tip). Scale bars: (**A**–**C**)—1 mm, (**D**–**H**)—0.5 mm, (**I**)—0.1 mm. All photos from specimen OSTR #7252, made by V. Plášek.

***Schistidium*** ***pulchrum*** H. H. Blom—Figure 3, and map: Figure 5 (spot 3).

Description: plants olivaceous, sometimes brownish, from 1.5 to 5 cm high; leaves erect, ovate-lanceolate, sharply keeled distally; capsule orange-brown, cylindric, from 0.8 to 1.3 mm, produced late spring to early summer.

Morphologically *S. pulchrum* resembles *S. apocarpum* (Hedw.) Bruch & Schimp., but differs in having smaller plants with shorter leaves, which are straight rather than curved, and subentire upper leaf margins. For details of the species morphology see [19,25].

Ecology: mainly on rocks in somewhat shaded habitats [19].

Specimen examined. W border of Xinjiang Uyghur Autonomous Region. 500 km SW of Urumqi; Tuo mu er peak National Reserve, Tuo mu er peak, ca. 2400 m a.s.l., 41.859722° N, 80.666389° E, leg. M. Sulayman s.n., 20 June 2018 (herb. XJU, OSTR #7252).

*Schistidium pulchrum* is a widespread circumpolar montane species throughout the northern Holarctic. It occurs in most Arctic archipelagoes, well studied for bryophytes throughout the Arctic and boreal zones, declining southward within the temperate zone. *Schistidium pulchrum* is a common species in the mountains of Siberia and the Russian Far East from Alai in the west to Sikhote-Alin in the east. It settles in various rocky ecotopes from the lower altitudinal belt up to 2700 m in Altai and 2000 m in the Republic of Buryatia [7,19,26].

**Figure 4:**
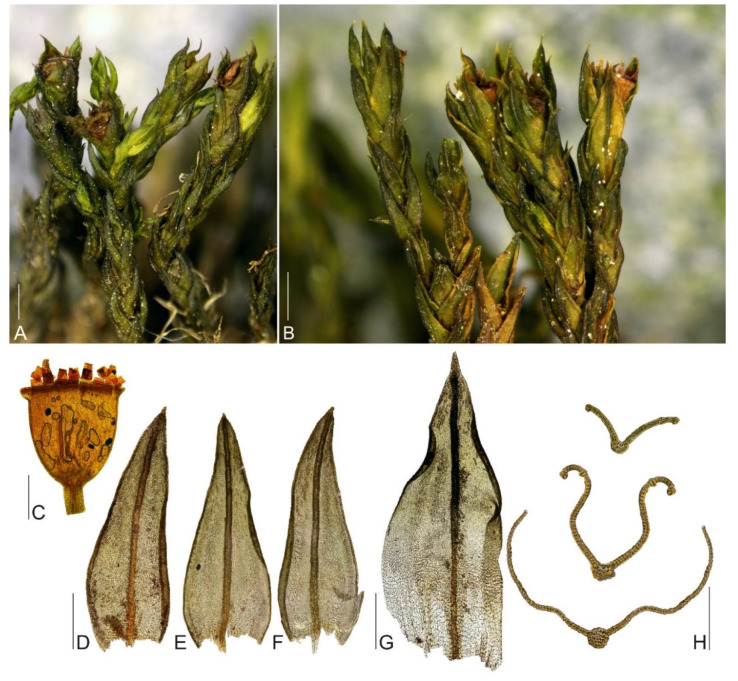
Macro and micro photographs of *Schistidium sibiricum*: (**A**,**B**) view on fertile plants; (**C**)—capsule; (**D**–**F**) leaves; (**G**) perichaetial leaf; (**H**) leaf cross-sections (from the base of the leaf to the tip). Scale bars: (**A**,**B**)—1 mm, **(C**–**G**)—0.5 mm, (**H**)—0.1 mm. All photos from specimen OSTR #7253, made by V. Plášek.

**Figure 5:**
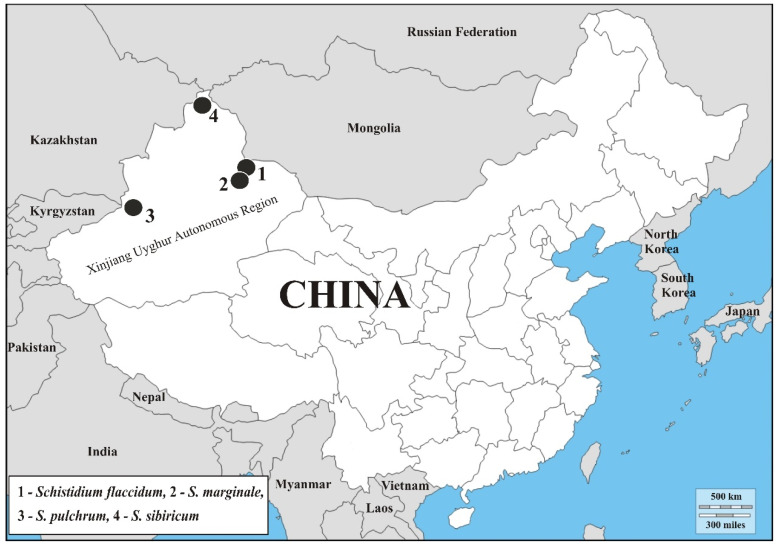
Map of China with distribution of newly recorded species.

***Schistidium*** ***sibiricum*** Ignatova & H.H. Blom—Figure 4, and map: Figure 5 (spot 4).

With its lacking hyaline hair point and costae short excurrent as a yellowish mucro, *Schistidium sibiricum* is quite distinct morphologically and may hardly be confused with the other *Schistidium* species except subatlantic *S. canadense* and Beringian *S. frahmianum*. This trait distinguishes it from *S. apocarpum*, which resembles *S. sibiricum* in general aspect, leaf shape, serrulate in upper leaf portion, peristome teeth strongly perforated, curved to twisted when dry [7]. *Schistidium canadense*, which for some time was considered a subspecies of *S. apocarpum* resembles *S. sibiricum*, even stronger, since it often has chlorophyllous leaf awns, and some older specimens of *S. sibiricum* were referred to *S. canadense/S. apocarpum* ssp. *Canadense.* However, molecular data showed that these species are not close phylogenetically and their resemblance has originated from convergent evolution [7,19].

Specimen examined. N part of Xinjiang Uyghur Autonomous Region, 600 km N of Urumqi, Altai mountains, Kanas Nature Reserve, ca. 1200 m a.s.l., 48.547571° N, 87.193903° E, leg. M. Sulayman s.n., 1 September 2006 (herb. XJU, OSTR #7253).

*Schistidium sibiricum* has mainly Asian distribution with few localities in the Ural Mountains, Kola Peninsula, south Finland and northern Norway. In Asia, it is a frequent species in the mountains of south Siberia and the southern part of the Russian Far East with several records from Kamchatka [7,19,26].

### 3.2. Checklist of Schistidium Species in China

At present, an occurrence of 15 species of the genus *Schistidium* is provably known in China. Four of them (marked in bold below) are newly recorded species. On the contrary, *S. striatum*, has been removed from the list due to the very unlikely occurrence in Chinese territory.

Accepted Taxa (newly recorded taxa are marked in bold):

*Schistidium apocarpum* (Hedw.) Bruch & Schimp.,

*Schistidium chenii* (S.H. Lin) T. Cao, C. Gao & J.C. Zhao,

***Schistidium* *flaccidum*** (De Not.) Ochyra,

*Schistidium ignatovae* C. Feng, X.L. Bai, J. Kou & W. Li,

*Schistidium lancifolium* (Kindb.) H.H. Blom,

*Schistidium liliputanum* (Müll. Hal.) Deguchi,

***Schistidium* *marginale*** H.H.Blom, Bednarek-Ochyra & Ochyra,

*Schistidium mucronatum* H.H. Blom, Shevock, D.G. Long & Ochyra,

***Schistidium* *pulchrum*** H. H. Blom,

*Schistidium riparium* H.H. Blom, Shevock, D.G. Long & Ochyra, 

*Schistidium rivulare* (Brid.) Podp., 

***Schistidium* *sibiricum*** Ignatova & H.H. Blom,

*Schistidium sinensiapocarpum* (Müll. Hal.) Ochyra, 

*Schistidium subconfertum* (Broth.) Deguchi, 

*Schistidium trichodon* (Brid.) Poelt).

Excluded Taxa:

*Schistidium strictum* (Turner) Loeske ex Mårtensson.

Note: The distribution of *S. strictum* in China is highly unlikely given that the area of occurrence of the species is quite far from the Chinese territory [1,19]. Previously this species was considered in a broader sense, including most of the currently recognized species of the genus with papillose leaf lamina, while *S. strictum* s.str. has suboceanic distribution in Europe and along the western coast of North America [25]. Special revision of the specimen is needed to assign it to one of the narrower circumscribed species; in Russia, *S. papillosum* Culm. is the most common species with papillose cells of leaf lamina, and most records of *S. strictum* were later referred to it. Southwards, it reaches the mountains of south Siberia and could occur in China as well.

### 3.3. Key to Schistidium Species in China

1a. Leaves broadly ovate to ovate-lanceolate, muticous21b. Leaves narrowly ovate-lanceolate to lanceolate32a. Leaves broadly ovate, strongly concave, rounded-obtuse at apex
*S. chenii*
2b. Leaves ovate-lanceolate, keeled above, acute at apex
*S. rivulare*
3a. Leaves without hyaline hair points or yellowed opaque leaf tip43b. Leaves at least with short hyaline hair points or yellowed opaque leaf tip54a. Leaves about 1 mm wide, slightly concave, peristome teeth longer (370–450 ųm) and strongly curved, twisted halfway around the axis
*S. mucronatum*
4b. Leaves narrower (0.45–0.8 mm), strongly concave, peristome teeth shorter (270–340 ųm) and straight, not twisted round the axis
*S. riparium*
5a. Leaves with yellowed opaque leaf tip
*S. sibiricum*
5b. Leaves with hyaline hair points66a. Peristome teeth vestigial76b. Peristome teeth well developed87a. Hyaline hair points short, oval in cross-section
*S. subconfertum*
7b. Hyaline hair points distinctive, wide and strongly flattened
*S. flaccidum*
8a. Hyaline hair points very long (up to 1.2 mm), broadly decurrent
*S. ignatovae*
8b. Hyaline hair points shorter, non or only narrowly decurrent99a. Peristome teeth linear, elongate, up to 700 µm long
*S. trichodon*
9b. Peristome teeth lanceolate, significantly shorter (up to 500 µm)1010a. Plants less than 1 cm high, capsules deeply immersed in perichaetial leaves
*S. liliputanum*
10b. Plants more than 1 cm high, capsules shallowly immersed in perichaetial leaves1111a. Leaf margin in upper part distally denticulate1211b. Leaf margin smooth along the entire length1312a. Upper leaves curved when dry, leaf margin in upper part sharply denticulate, costa coarsely and highly papillose on abaxial side
*S. lancifolium*
12b. Upper leaves straight erect-patent when dry, leaf margin in upper part bluntlydenticulate, costa smooth or with low papillae on abaxial side
*S. apocarpum*
13a. Leaf margins in upper parts in 2–3 rows, 4-stratose, costa flattened in upper part

*S. marginale*
13b. Leaf margins in upper part in 1 row, 2-stratose, costa not flattened in upper part
1414a. Hair points densely and sharply toothed, costa papillose on abaxial side

*S. sinensiapocarpum*
14b. Hair points smooth or faintly toothed, costa smooth on abaxial side
*S. pulchrum*


## 4. Conclusions

Four newly found species of *Schistidium* are reported from China. An occurrence of 15 species is recently known in the country. Given that, *Schistidium* is a critical genus that is still being investigated in detail, we expect further interesting results and findings in the near future.

## Data Availability

Not applicable.
